# Advancements in Tobacco (*Nicotiana tabacum* L.) Seed Oils for Biodiesel Production

**DOI:** 10.3389/fchem.2021.834936

**Published:** 2022-01-18

**Authors:** Shengjiang Wu, Chuanchuan Gao, Hu Pan, Kesu Wei, Delun Li, Kai Cai, Heng Zhang

**Affiliations:** ^1^ Guizhou Academy of Tobacco Science, Upland Flue-cured Tobacco Quality and Ecology Key Laboratory, CNTC, Guiyang, China; ^2^ Guizhou Tobacco Quality Supervision and Test Station, Guiyang, China; ^3^ College of Biological, Chemical Science and Engineering, Jiaxing University, Jiaxing, China; ^4^ State Key Laboratory Breeding Base of Green Pesticide and Agricultural Bioengineering, Key Laboratory of Green Pesticide and Agricultural Bioengineering, Ministry of Education, State-Local Joint Laboratory for Comprehensive Utilization of Biomass, Center for Research and Development of Fine Chemicals, Guizhou University, Guiyang, China

**Keywords:** biodiesel, tobacco seed, inedible oilseed crop, biodiesel property, biotechnology

## Abstract

With the increasing demand for fossil fuels, decreasing fossil fuel reserves and deteriorating global environment, humanity urgently need to explore new clean and renewable energy to replace fossil fuel resources. Biodiesel, as an environmentally friendly fuel that has attracted considerable attention because of its renewable, biodegradable, and non-toxic superiority, seems to be a solution for future fuel production. Tobacco (*Nicotiana tabacum* L.), an industrial crop, is traditionally used for manufacturing cigarettes. More importantly, tobacco seed is also widely being deemed as a typical inedible oilseed crop for the production of second-generation biodiesel. Advancements in raw material and enhanced production methods are currently needed for the large-scale and sustainable production of biodiesel. To this end, this study reviews various aspects of extraction and transesterification methods, genetic and agricultural modification, and properties and application of tobacco biodiesel, while discussing the key problems in tobacco biodiesel production and application. Besides, the proposals of new ways or methods for producing biodiesel from tobacco crops are presented. Based on this review, we anticipate that this can further promote the development and application of biodiesel from tobacco seed oil by increasing the availability and reducing the costs of extraction, transesterification, and purification methods, cultivating new varieties or transgenic lines with high oilseed contents, formulating scientific agricultural norms and policies, and improving the environmental properties of biodiesel.

## Introduction

**FIGURE 1:**
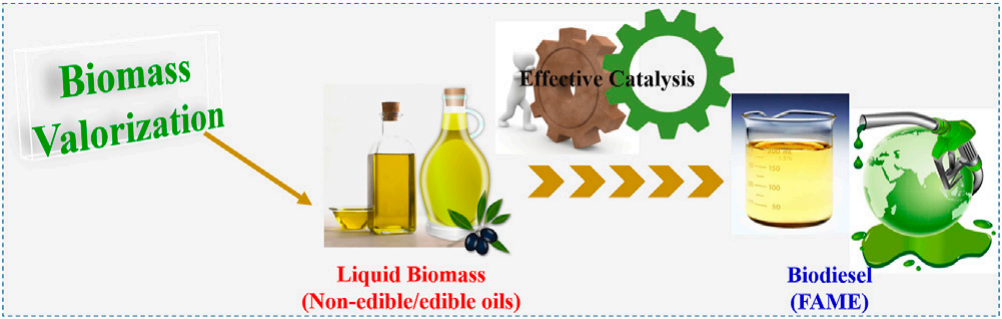
Schematic illustration of catalytic conversion of liquid biomass into biodiesel.

The industrial revolution results in the widespread use of fossil fuels, which constitute 80% of the energy supply. Additionally, various fossil fuel derivatives are also widely used in the production of industrial and agricultural products. In the past 2 decades, global fossil fuel consumption has continued to grow by 48.7%. According to the reports in 2019, the corresponding consumption reached 136,761 TW-hours (TWh), and oil, natural gas and coal were 53,620, 39,292 and 43,849, respectively (Smil, 2017). The global carbon balance has been broken with massive amounts of CO_2_ emitted from the use of fossil fuels. The carbon surplus of the atmosphere is the major driving force for the modern global environment and climate change issues such as global warming, extreme weather, etc. (Pao and Tsai, 2010; Pan et al., 2020). To mitigate the negative consequences of excess CO_2_ emissions, biofuels derived from waste have been recognized as a substitute for the traditional fossil fuels (Kim et al., 2019). They are carbon neutral, inexpensive and an environmentally friendly and renewable source of energy (Hill et al., 2006). Among the various types of biofuels, biodiesel has been characterized by lower agricultural inputs and more efficient conversion (Hill et al., 2006). Biodiesel has been widely commercialized due to its relatively mature production technology, simple and universal platform (Figure 1) (Liu et al., 2019). It is inferred that the global production of biodiesel will reach 46 billion liters in 2025 (Zhang et al., 2017).

Although biodiesel brings many benefits, the key basis of biodiesel production is feedstocks, and feedstocks are determined to play the most important role in the biodiesel value chain. The feedstock should fulfill three main requirements: low production costs, large-scale production and appropriate fatty acid content/composition for biodiesel (Mishra and Goswami, 2018). Moreover, their oil content and composition are highly important indicators, such as the feedstocks rich in monounsaturated fatty acids (MUFAs) are desirable for biodiesel but the composition of the saturated fatty acids (SFAs) is also shown to be of great importance (Stansell, et al., 2012). According to the source of feedstocks, we can categorize them into three biodiesel generations. First-generation biodiesel is currently being widely produced and is extracted from various feedstocks, such as edible rapeseed oil, sunflower oil, palm oil, soybeans, and animal fats. Second- and third-generation biodiesels, only a few of which are close to large-scale commercialization, are extracted from inedible lipid feedstocks such as terrestrial biomass (i.e., breeding crops and transgenic crops) and aquatic biomass (i.e., microalgae and macroalgae) (Vauhkonen et al., 2009; Chung et al., 2020; Mizik and Gyarmati, 2021). However, biodiesel produced from edible oils is more expensive than conventional fossil diesel. Biodiesel has over double the price of diesel fuel. The major economic factor to consider for input costs of biodiesel production is the feedstock, which is about 80% of the total operating cost (Demirbas, 2007). On the other hand, food shortages may occur due to the use of large amounts of edible oils in producing biodiesel, especially in poor countries. These drawbacks can be overcome by using inedible oils for biodiesel production like *Aleurites trisperma*, bitter almond, *Brucea javanica* seeds, Argemone Mexicana, wild mustard (*Brassica Juncea L.*) seed, and tobacco (*Nicotiana tabacum* L.) seed (Arumugam et al., 2017; Muthukumaran et al., 2017; Jain, 2019; Wang et al., 2019; Adepoju, 2020; Tlili et al., 2020). Generally, tobacco, *Nicotiana tabacum*, which is an annual plant in the Solanaceae family with leaves harvested to manufacture cigarettes, is widespread in China and North and South America, and the leaves are commonly harvested. In recent years, tobacco has been cultivated in approximately 130 countries and covered almost 3.4 million hectares in fields, wherein China alone provides over 40% of the world’s production (García-Martínez et al., 2017; Berbe and Matyka, 2020; Wu et al., 2020). It is reported that the *N. tabacum* varieties with the highest seed yields reach 1,171 kg per hectare, which corresponds to 432.9 kg of oil based on an effective oil biosynthesis mechanism for tobacco (Zdremnan and Zdremnan, 2006; García-Martínez et al., 2017). The oil content of tobacco seed often ranges from 30 to 43 wt% and consists mainly of unsaturated fatty acids (Giannelos et al., 2002; Banković-Ilić et al., 2012; Khan et al., 2014; Hu et al., 2020; Rajan et al., 2021). The major fatty acids in tobacco seed oil are stearic acid (2.1–3.3%), palmitic acid (8.72–12.3%), oleic acid (9.97–13.49%), and linoleic acid (64.38–79.0%), respectively (Koiwai et al., 1983; Mukhtar et al., 2007; Tian et al., 2020). As a kind of second-generation biodiesel, tobacco-based biodiesel production is very promising. Therefore, in this review, we elucidate the extraction and transesterification methods, genetic modification, properties and application, and agricultural strategies applied in tobacco biodiesel production, and then discuss the key problems and prospects for biodiesel production from tobacco.

## Extraction Methods of Tobacco Seed Oil for Biodiesel Production

The tobacco seed oil has been traditionally extracted by mechanical pressing and solvent extraction, mainly with nonpolar solvents. Mechanical pressing, a relatively environmentally friendly extraction method, has been widely applied for the production of high-quality edible oils (Sannino et al., 2017). The whole process includes sample preparation and mechanical pressing stages followed by a solvent extraction procedure to recover the oil located in the press cake (Geow et al., 2021). Seed preparation involving preheating and microwaving destroy or soften the cellular structure of tobacco seeds, which significantly increases the oil yield (Sannino et al., 2017). The pressing equipment parameters including feeding rate, restriction dye diameter, temperature and rotation speed, affect the tobacco seed oil yield. The evaluation of extraction methods in the literature based on the oil yield provided by Soxhlet or accelerated solvent extraction (ASE) due to high oil recovery.

Oil extraction yield obtained from mechanical pressing were usually about 80% (*w/w*) of ASE, but Stanisavljević et al. reported a higher oil extraction yield, which may be related to pressing equipment type (Stanisavljević et al., 2009; Faugno et al., 2016; Sannino et al., 2017). The characteristics of several common methods used in tobacco seed oil extraction is shown in Table 1.

**TABLE 1:** Characteristics of several common methods used in tobacco seed oil extraction.

Methods	Advantages	Disadvantages	Extraction efficiency	Key parameters
Mechanical pressing	Economic, environment friendly, high quality oil and throughput, simple crude oil separation	Lower oil recovery, high power consumption, partial oil left in seed cake	80.28% Sannino et al. (2017).^a^, 79.47% Faugno et al. (2016).^a^, comparable to Soxhlet extraction Stanisavljević et al. (2009)	Machine type, feeding rate, restriction dye diameter, temperature, rotation speed, seed pretreatment
Soxhlet extraction	High oil recovery, simple operation	Time consuming, consuming organic solvent, complex crude oil separation	100% Stanisavljević et al. (2007b) and Stanisavljević et al. (2009)	Extraction solvent, temperature and time, seed-to-solvent ratio, seed pretreatment
Ultrasonic extraction	Short extraction time, simple and moderate operation	Lower oil recovery, consuming organic solvent, specific equipment, complex crude oil separation	45.81–72.12% Stanisavljević et al. (2009)	Ultrasonic power, extraction solvent, temperature and time, seed-to-solvent ratio, seed pretreatment
			78.20% Stanisavljević et al. (2007b)	
Accelerated solvent extraction	Short extraction time, high oil recovery, consuming less organic solvent	High operating cost, specific equipment	100% Faugno et al. (2016); Sannino et al. (2017).^a^	Extraction solvent, temperature, time and pressure, static cycle, seed pretreatment
Supercritical fluid extraction	Environment friendly, high quality oil, simple crude oil separation	Lower oil recovery, high operating cost, specific equipment	56.49–78.85% Majdi et al. (2012)	Extraction pressure, temperature and time, modifier type, seed pretreatment
Maceration	Short extraction time, simple and moderate operation	Lowest oil recovery, consuming organic solvent, complex crude oil separation	56.91–57.55% Stanisavljević et al. (2009)	Extraction solvent, temperature and time, seed pretreatment

a The ratio of crude oil content obtained from different extraction methods compared to ASE.

“Seed pretreatment”-Seed drying, seed size reduction, seed preheating, seed microwaving, etc.

Soxhlet extraction is a classical method for extracting tobacco seed oil with high extraction efficiency (Maestri and Guzmán, 1995; Zlatanov, et al., 2007). Compared with Soxhlet extraction, ASE with a similar oil yield consumed less solvent and time (Faugno et al., 2016; Sannino et al., 2017). However, the milder extraction operations, such as ultrasonic extraction and maceration, showed a lower extraction efficiency (Stanisavljević et al., 2007b and 2009). The solvent extraction efficiency from tobacco seed oil depends on the comminution of the seed, the solvent, the seed-to-solvent ratio, and the extraction time and temperature. Specifically, milling greatly improves extraction efficiency by breaking down tobacco seed cells and augmenting the interfacial area for mass transfer. The oil yield obtained from ground tobacco seeds was shown to be 10 times that of native seeds during ultrasonic extraction, even though the oil yield of tobacco seeds repeatedly grounded was 6 times higher than that of native seed extracted by mechanical pressing (Stanisavljević et al., 2009). Supercritical fluid extraction was also used to extract tobacco seed oil with high recovery and without any residual organic solvents (Stanisavljević et al., 2007b; Majdi et al., 2012; Ashraf-Khorassani et al., 2015). It is worth emphasizing that pressure is the most important factor in supercritical fluid extraction. These extractions of tobacco seed oil mainly focus on laboratory- or small-scale operations. However, application at the industrial scale is still limited to date. Future research should study and develop more environmentally friendly extraction methods suitable for the large-scale production of tobacco seed oil.

## Transesterification of Biodiesel in Tobacco Seed Oil

**FIGURE 2:**
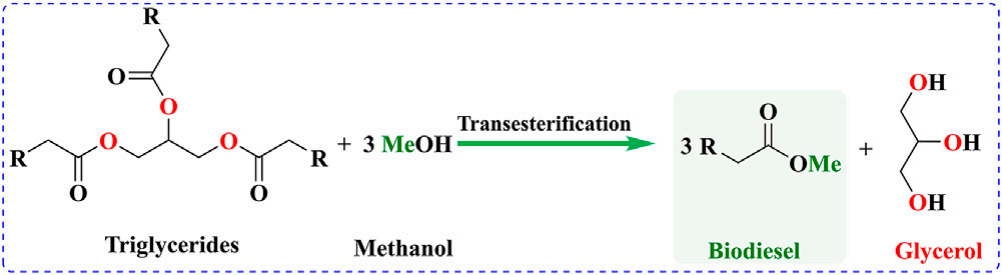
General chemical reaction for the transesterification of triglycerides.

Also known as alcoholysis, transesterification is a process involving the breakage of old ester bonds and the formation of new ester bonds with another alcohol. This process is similar to the mechanism of hydrolysis (Srivastava and Prasad, 2000). Transesterification reactions have been widely applied to convert triglycerides to fatty acid methyl esters or fatty acid ether esters with reduced oil viscosity. The typical reaction with common methanol is shown in Figure 2 and is also called methanolysis. The transesterification reactions of the process are reversible, and the presence of a suitable catalyst can accelerate the forward conversion reaction. Although acid and enzyme have also been applied in transesterification, base is the most commonly used catalyst and it mainly consists of three steps. These consist of alkoxide formation, nucleophilic attack at the carbonyl carbon, and the formation of the alkyl ester, which needs to be repeated three times for TGAs. The mechanism of acid-catalyzed transesterification is a little different and consists of carbocation formation, nucleophilic attack of the alcohol, and the formation of the alkyl ester (Ma and Hanna, 1999; Meher et al., 2006). The transesterification process determines the cost and complexity of tobacco seed biodiesel production.

Transesterification efficiency is affected by various critical factors, such as the influence of free fatty acids (FFAs) and water, type and amount of catalyst, the molar ratio of alcohol to oil and type of alcohol, transesterification time and temperature, uniformity of mixing, and whether or not organic co-solvents are used, depending upon reaction conditions. The catalyst chosen is ultimately critical and determines the degree of influence by other factors (Meher et al., 2006). Therefore, research on high-efficiency, low-cost and recyclable catalysts is of high importance.

Transesterification for biodiesel production can be carried out catalytically and non-catalytically under supercritical conditions and enzyme-catalyzed reactions (Leung et al., 2010). Catalytic reactions can also be divided into homogeneous/heterogeneous alkali-catalyzed and homogeneous/heterogeneous acid-catalyzed reactions (Avhad and Marchetti, 2015). The application of transesterification with tobacco seeds is shown in Table 2. Generally, the content of FFAs in tobacco seeds is approximately 6% (Stanisavljević et al., 2007a). This high level would result in saponification and then the difficulty in transesterification because the FFA level exceeds 2% (Anguebes-Franseschi et al., 2016). To decrease acid levels, pre-esterification with homogeneous acid could be performed. FFA content was shown to decrease to approximately 1.00% after pre-esterification. This content met the standards for the alkali-catalyzed transesterification process into biodiesel (Murmu et al., 2017). Compared with other catalysts, alkali catalysts are more commonly used with tobacco seed oil. The common homogeneous and heterogeneous alkalis reported in the literature are KOH, NaOH, CH_3_ONa, and KHCO_3_/Al_2_O_3_ (Veljković et al., 2006; Parlak et al., 2009; Anastopoulos et al., 2011; Waheed et al., 2015; Hariram and Rajan, 2016; Karabas et al., 2016, 2019; Motojesi et al., 2017; Fornasier et al., 2018; Samuel et al., 2020; Santoso et al., 2021). Due to their low costs and high catalytic capacities, NaOH and KOH are the most commonly used homogeneous alkali catalysts. Moreover, KHCO_3_/Al_2_O_3_, a heterogeneous alkali catalyst, can be rapidly separated from the reaction solution with centrifugation or filtration in the subsequent purification process. Moreover, this solid heterogeneous catalyst can simultaneously catalyze transesterification and esterification reactions with little impact from the high contents of FFAs. Thus, these heterogeneous catalysts are very suitable for application with feedstocks containing high FFA contents. However, this reaction often requires a relatively long time because the uniformity of the three-phase transesterification reaction is poor (Kulkarni et al., 2006; Liu et al., 2008). The non-catalytic supercritical method with methanol is also applied in tobacco seed oil conversion to biodiesel. The supercritical transesterification method often requires much higher temperature and pressure in a sealed reactor with an external heater. The transesterification reaction starts during the heating and pressurization period. Compared with the conventional alkali-catalyzed method, this process has advantages in terms of environmental friendliness and ease of purification but requires more energy consumption due to the high temperature and pressure utilized (García-Martínez et al., 2017). Recently, enzyme catalysts for biodiesel production have become increasingly attractive since they do not undergo saponification, and the subsequent purification process is simple. However, these catalysts are less often used commercially because of relatively long transesterification times and high costs (Moazeni et al., 2019).

**TABLE 2:** Catalysts for the transesterification of tobacco seed oil.

Catalyst types	Catalyst	Oil to MeOH ratio	Time/min	Catalyst amount, *w/w*	Temp/°C	Conversion efficiency, %	Ref
Homogeneous acid/alkali	H_2_SO_4_	7:1	120	8.1%	65	84.6	Santoso et al. (2021)
	KOH	6:1	10	1.1%			
Homogeneous acid/alkali	H_2_SO_4_	1:7.5	60	2.5%	65	90.2	Waheed et al. (2015)
	KOH	1:6.09	78	1.1%			
Homogeneous acid/alkali	H_2_SO_4_	5:1	Total	2.0%	60	92.0	Hariram and Rajan, (2016)
	NaOH	5:1	100	0.7%			
Homogeneous acid/alkali	H_2_SO_4_	1:18	25–50	2.0%	60	91.0	Veljković et al. (2006)
	KOH	1:6	20–30	1.0%			
Homogeneous acid/alkali	H_2_SO_4_	1:18	60	1.0%	50–65	82.0–96.0	Motojesi et al. (2017)
	KOH	1:18		1.0%			
or ash	Cocoa pod ash and rice husk ash	1:6		Ash for 2.0%			
Homogeneous alkali	KOH	4:1	—	1.5%	50	—	Karabas et al., 2018
Homogeneous alkali	KOH	5:1	—	1.0%	60	—	Karabas et al. (2016)
Homogeneous alkali	KOH	1:6	80	1.1%	60	>95.0	Samuel et al. (2020)
Homogeneous alkali	KOH	10:1	5	1.0%	50–60	98.0	Parlak et al. (2009)
Homogeneous alkali	CH_3_ONa	1:4–1:8	20	2.0%	70	97.0	Fornasier et al. (2018)
Heterogeneous Acid/alkali	KHCO_3_/Al_2_O_3_	1:8	150	3.0% w/w	60	97.1	Anastopoulos et al. (2011)
Noncatalytic with supercritical MeOH	—	1:43	90	—	303	92.8	García-Martínez et al. (2017)

## Role of Biotechnology in Biodiesel Production in Tobacco Seed Oil

Biotechnology is an important method for improving tobacco seed oil production and accumulation. In recent years, fatty acid and TAG accumulation in tobacco seeds has become a promising way to meet the increasing demand for renewable biofuels (Andrianov et al., 2010; Li et al., 2010; Hernández et al., 2012). The first step of fatty acid synthesis involves acetyl-CoA carboxylase (ACCase) catalysis of the carboxylation of acetyl-CoA to malonyl-CoA (Alonso et al., 2010; Fan et al., 2013). Phosphoenolpyruvate carboxylases (PEPCs) play important roles in fatty acid biosynthesis in seeds of oil plants by regulating carbon partitioning (Fan et al., 2013). During seed oil production and accumulation in oil plants, the increased mRNA level of PEPC indicates a potential role in partitioning carbohydrates to fatty acid biosynthesis (Azocar et al., 2010; Fan et al., 2013). Overexpression of *JcPEPC1* in tobacco results in an increase in PEPC activity and fatty acid content, proving its important role in seed oil biosynthesis (Fan et al., 2013).

During TAG accumulation, diacylglycerol acyltransferases (DGATs) are the final enzymes employed in TAG biosynthesis, and they play a critical role in plant oleaginous seed production (Xu et al., 2014). In particular, overexpression of DGATs was shown to increase the accumulation of TAG in plant seeds (Li et al., 2010; Xu et al., 2014). To date, the *DGAT1*, *DGAT2* and *DGAT3* gene families that encode the DGAT enzymes have been identified in plants (Li et al., 2010; Hernández et al., 2012; Xu et al., 2014). Xu et al. (2014) reported that overexpression of *JcDGAT1* and *JcDGAT2* from *J. curcas* resulted in increases in seed oil contents of 32.8 and 31.8%, respectively, in transgenic tobacco.

Most recently, the CRISPR-Cas9 system has emerged as a programmable and versatile tool for genome editing in a wide variety of organisms (Tian et al., 2020 and 2021). Altering the fatty acid metabolic profile by raising the oleic acid content improves the properties of the biodiesel produced and accumulated from tobacco seed oil. Tobacco *fad2–2* mutant seeds based on CRISPR-Cas9 gene-editing technology showed a sharp increase in oleic acid content from 11% to over 79%, whereas the linoleic acid content dropped from 72 to 7% (Tian et al., 2020). Additionally, lipid accumulation in tobacco seeds was dramatically enhanced by approximately 18 and 15% in two targeted knockout mutant lines, of which the *NtAn1* gene of the *TT8* homolog was created by CRISPR-Cas9 (Tian et al., 2021). Yang et al. (2006) employed a partial coding sequence of a microsomal ω-6 fatty acid desaturase gene (*FAD2*) associated with the first step in polyunsaturated fatty acid biosynthesis to make an hpRNA-producing construct to more specifically silence the endogenous *FAD2* gene, thereby realizing a significant increase in the oleic acid level in tobacco seed lipids. In addition, Zhang L. et al. (2016) stated that the oleic acid content relative to the content of total seed lipids of transformants was drastically decreased in FAD2-silenced tobacco at a low temperature, and there was a corresponding increase in the content of polyunsaturated fatty acid. The reduction in *DGAT1* transcript levels in transgenic tobacco mediated by hpRNA is correlated with a decrease in oil content of 9–49% in mature seeds of transgenic lines (Zhang et al., 2005).

**FIGURE 3:**
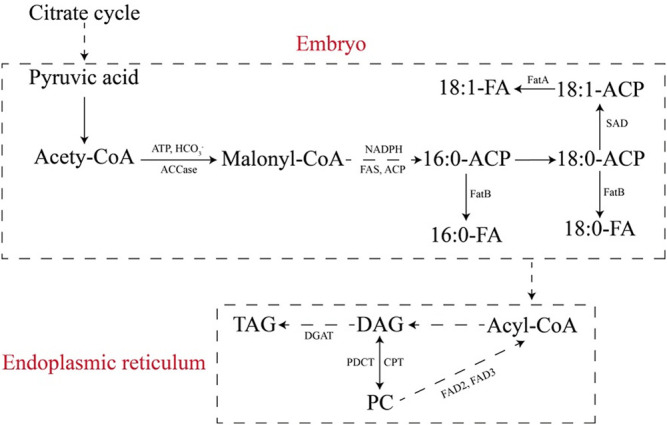
Fatty acid and TAG metabolic pathways in tobacco seeds. ACCase, acetyl-CoA carboxylase; FAS, fatty acid synthase; ACP, acyl carrier protein; FA, fatty acid; FatA/B, acyl-ACP thioesterases; DAG, diacyl-glycerol; TAG, triacylglycerol; PC, phosphatidylcholine; SAD, stearoyl-ACP desaturase; DGAT, diacylglycerol acyltransferase; PDCT, phosphatidylcholine:diacylglycerol cholinephosphotransferase; CPT, CDP-choline:1,2-diacylglycerol cholinephosphotransferase; FAD2, oleoyl desaturase; FAD3, linoleoyl desaturase (Zhang, et al., 2016b; Zhou et al., 2019).

Identifying important regulatory factors involved in tobacco seed oil biosynthesis may enhance our understanding of the process of oil biosynthesis and improve strategies for increasing oil production. It has also been reported that several transcription factors, such as *LEC1*, *LEC2*, and *FUS3*, are associated with embryo development and seed oil biosynthesis (Chen et al., 2014; Zhang M. et al., 2016). However, many genes, proteins, regulatory factors, and multiple pathways are associated with the processes of tobacco seed oil biosynthesis and accumulation (Figure 3), and the molecular mechanisms of fatty acid and TAG biosyntheses are not fully understood (Zhang M. et al., 2016; Zhou et al., 2019).

## Influencing Factors for Biodiesel Production During Tobacco Seed Oil Accumulation

During seed development, the contents of TAG, diacylglycerol, and sterol esters were shown to increase, while those of phosphatidylcholine, phosphatidylethanolamine, phosphatidylinositol, phosphatidylglycerol, monogalactosyldiacylglycerol, digalactosyldiacylglycerol, and sulfoquinovosyldiacylglycerol were shown to decrease, suggesting that the proportions of linoleate and linolenate decreased with concomitant increased proportions of stearate and oleate in the phospholipids (Koiwai et al., 1982). The contents of tobacco seed fatty acids in ovaries grown in the greenhouse were more than four-fold higher than those in mature ovaries cultured *in vitro* because of many cultural factors, such as mineral and sugar concentrations, plant hormone interactions, oxygen concentration, and culture temperatures (Toshiake et al., 2006). Lin et al. (2020) were able to increase the seed and oil yields and modify the fatty acid composition of tobacco plants by enhancing the level of nitrogen applied. However, the effects of many influencing factors (such as the ecological environment, heredity, exogenous hormones, etc.) and agricultural strategies (e.g., Fertilization, irrigation, weeding, diseases and pests control) on tobacco seed oil biosynthesis and accumulation associated with biodiesel production have not been revealed.

## Properties and Application of Biodiesel From Tobacco Seed Oil

Tobacco seed oil cannot be used directly in diesel engines, primarily due to its high viscosity and low heat content (Sharma et al., 2008). Transesterification reaction converts triglycerides into methyl esters to meet the specifications of biodiesel standards. Many countries have implemented national standards to promote biodiesel quality and regulate biodiesel production (Yang et al., 2016). Generally, the evaluation of biodiesel properties is based on ASTM D6751-2015c and BS EN 14214-2012 + A1-2019 standards (BSI, 2019; ASTM D6751-20a, 2020). Biodiesel is characterized by considering its physicalchemical and fuel properties, such as density, kinematic viscosity, cloud and pour point, energy content, cetane index (Ashraful et al., 2014). It is worth emphasizing that carbon chain length and saturation of fatty acids are important factors affecting the properties of biodiesel (Knothe, 2005). Additionally, the fluidity of biodiesel is related to density, kinematic viscosity, and cloud and pour point. The energy content and cetane index influence the production of torque and power. The flashpoint and ignition point are important parameters for evaluating the safety of biodiesel. Biodiesel has relatively low contents of ash, sulfur, and carbon residue; thus, the amount of pollutants emitted is reduced. Biodiesel usually has limited stability and is likely to form polymers that may block the fuel supply system. The amounts of monoglycerides, diglycerides, triglycerides, and free and total glycerine are important indicators of biodiesel purity. The metal ion residues (Na^+^, K^+^, Ca^2+^, Mg^2+^) introduced by catalysts could cause deposition and wear of engine components. Water and methanol contents and acid values may also accelerate erosion of the injection system and increase carbon deposition in engines. An evaluation of the important properties of tobacco seed oil biodiesel (TSOBD) is shown in Table 3 (Veljković et al., 2006; Anastopoulos et al., 2011; Usta et al., 2011; Parlak et al., 2012; Rao B. S. et al., 2013, 2013c; Waheed et al., 2015; Hariram and Rajan, 2016; Guntur and Prasanthi, 2018; Karabas et al., 2019; He et al., 2021).

**TABLE 3:** Comparisons of the properties of TSOBD under different standards.

Parameters	Recommended range			References											
	EN 14214-2012	ASTM D6751-15a	GB25199 -2017	He et al. (2021)	Veljković et al. (2006)	Usta, (2005)	Anastopoulos et al. (2011)	Parlak et al. (2012)		Rao et al. (2013c)	Waheed et al. (2015)	Rao et al. (2013a)	Hariram and Rajan, (2016)	Guntur and Prasanthi, (2018)	Karabas et al. (2019)
								KOH	NaOH						
Fatty acid methyl esters content (%)	>96.5	—	>96.5	98.15	—	98.6	—	96.5	97	—	—	—	—	—	96.5
Density (kg/m^3^)	860–900	—	820–900	887	882	888.5	917.5	880	860	870	890	870	921	870	880
Flash point (°C)	>101	>130	>130	—	—	165.4	220	>100	152	174	126	—	262	174	135
Water and sediment volume (%)	—	<0.05	—	—	—	—	—	—	—	—	—	—	—	—	—
Sulfur content (mg/kg)	<10	S15 < 15, S50 < 500	S50* < 50, S10* < 10	6	—	8	9	0	0	0	—	—	—	—	—
Carbon residue (%)	<0.30	<0.05	<0.05	—	—	0.029	0.086	0.17	0.17	—	—	—	—	—	0.17
Cetane index	>51	>47	S50* > 49, S10* > 51	51	—	51.6	—	54.5	49	55	—	52	26	35	54.5
Sulfated ash (%)	<0.02	<0.02	<0.02	—	—	0.0004	—	—	—	—	—	—	—	—	—
Water content (mg/kg)	<500	—	<500	368	—	354	570	300	400	—	—	—	—	—	—
Viscosity (mm^2^/s)	3.50–5.00	1.9–6.0	1.9–6.0	3.5	5.2	4.23	27.7	4.88	3.5	4.2	3.87	13.7	3.5	4.12	4.88
Total contamination (mg/kg)	<24	—	—	—	—	23.95	—	20	23	—	—	—	—	—	20
Copper strip corrosion (rating)	Class 1	Class 3	Class 1	—	—	Class 1	—	Class 1	Class 1	—	—	—	—	—	Class 1
Oxidation stability (h)	>8	>3	>6	—	—	0.8	—	—	—	—	—	—	—	—	—
Acid value (mg KOH/g)	<0.5	<0.5	<0.5	0.45	0.66	0.30	0.48	—	—	—	0.42	—	0.56	0.49	0.25
Iodine value (g I_2_/100)	<120	—	—	112	110	136	135	122	118	—	—	—	139	110	122
Linolenic acid methyl ester (%)	<12	—	—	—	—	0.759	—	—	—	—	—	—	—	—	—
Polyunsaturated (≥4 double bonds) methyl esters (%)	<1	—	—	—	—	<0.1	—	—	—	—	—	—	—	—	—
Methanol content (%)	<0.20	<0.20	—	—	—	<0.01	—	0.20	0.18	—	—	—	—	—	—
Monoglyceride content (%)	<0.70	Grade No. 1B < 0.40	<0.80	—	—	0.54	—	0.29	<0.29	—	—	—	—	—	—
Diglyceride content (%)	<0.2	—	—	—		0.13	—	0.20	<0.05	—	—	—	—	—	—
Triglyceride content (%)	<0.2	—	—	—	—	0.17	—	0.11	0.11	—	—	—	—	—	—
Free glycerine (%)	<0.02	<0.02	<0.02	—	—	0.002	—	—	—	—	—	—	—	—	—
Total glycerine (%)	<0.25	<0.24	<0.24	—	—	0.23	—	0.02	0.02	—	—	—	—	—	0.02
Phosphorus content (mg/kg)	<4	<10	<10	—	—	4	—	4	<4	—	—	—	—	—	—
Group I metals (Na + K) (mg/kg)	<5	<5	<5	—	—	<5	—	—	—	—	—	—	—	—	—
Group II metals (Ca + Mg) (mg/kg)	<5	<5	<5	—	—	<5	—	—	—	—	—	—	—	—	—
Cold filter plug point (°C)	**—**	—	—	—	—	-5	—	−7	−10	—	—	—	—	—	—
Gross calorific value (cal/g)	—	—	—	9,510	—	—	—	9,355	9,560	8,958	—	9,078	9,510	7,764	9,355
Pour point (°C)	—	—	—	—	—	—	—	−6	−12	—	—	—	—	—	-6
Distillation temperature (°C)	—	<360	—	—	—	—	—	—	—	—	—	—	—	—	—

“—“-parameters that are not regulated in standards or tests in the literature.

“S15”-a grade of biodiesel meeting ASTM, Specification D6751 and having a sulfur specification of 15 ppm maximum.

“S50”-a grade of biodiesel meeting ASTM, Specification D6751 and having a sulfur specification of 500 ppm maximum.

“S10*”-a grade of biodiesel meeting GB25199-2017 and having a sulfur specification of 10 ppm maximum.

“S50*”-a grade of biodiesel meeting GB25199-2017 and having a sulfur specification of 50 ppm maximum.

“Grade No. 1B”-A special purpose biodiesel blend stock intended for use in middle distillate fuel applications that can be sensitive to the presence of partially reacted glycerides, including those applications requiring good low temperature operability.

“NaOH, and KOH”-Catalysts used in transesterification reaction.

Generally, the flashpoint, density, acid value, sulfur content, and carbon residue of TSOBD are within or near the ranges recommended by European standards. Some parameters, such as oxidation stability, cetane index, iodine value, and cold filter plugging point, are correlated with the methyl ester content of TSOBD (Ramos et al., 2008). Variations in these indexes may be explained by the different characteristics of tobacco seeds and the whole biodiesel production process. Tobacco seed oil is rich in unsaturated fatty acids; thus, TSOBD has a higher iodine value and unsatisfactory oxidation stability (Usta et al., 2011), which can also be altered by the type and amount of catalyst used in the transesterification reaction (Parlak et al., 2012). The addition of antioxidants can enhance oxidation stability. TSOBD has a lower energy content and cetane index than fossil diesel, which can be improved by blending with other types of biodiesels or pure diesel (Usta, 2005; Anastopoulos et al., 2011). TSOBD addition can also improve the lubrication of engine fuel systems and extend the operating life of system components (Cesur et al., 2014).

Brake power, brake thermal efficiency (BTE), and brake special fuel consumption (BSFC) are used to evaluate the fuel characteristics of biodiesel (Ashraful et al., 2014). TSOBD blends are compatible with pure diesel uses and can increase combustion efficiency and show relatively high brake power and BTE and low BSFC in most cases. On the other hand, TSOBD blends in diesel engines can reduce CO and SO_2_ emissions while slightly increasing NO_x_ emissions (Usta, 2005; He et al., 2021). The TSOBD mixing ratio (Parlak et al., 2012), injection opening pressure (Hountalas et al., 2003), injection time (Teoh et al., 2015), engine load (He et al., 2021), engine speed (Parlak et al., 2013), engine type (Rao et al., 2013c), and compression ratio (Guntur and Prasanthi, 2018) affect TSOBD blend properties and pollutant emissions. It is worth noting that a fuel blend with a low percentage of TSOBD is more efficient at a higher load than at a partial load (Usta, 2005). Compared to diesel, NO_x_ emissions are slightly increased due to high temperatures and oxygen levels in the cylinder at relatively high load, but there is no significant difference in NO_x_ emissions at partial load. In addition, Sharma et al. (2020) examined the impact of engine input parameters on the engine performance using the response surface methodology approach. Low heat rejection diesel engines with relatively high capacities to handle fuels with low calorific values can enhance TSOBD fuel characteristics while also producing more NO_x_ emissions than conventional engines (Rao N. V. et al., 2013). The NO_x_ emissions of variable compression ratio diesel engine using TSOBD decreased nearly 50% at 16:1 compression ratio compared to 18:1 compression ratio at half and full load (Guntur and Prasanthi, 2018). In addition, catalysts that convert NO_x_ into N_2_, such as traditional three-way and new developed zeolite-based catalyst, can be introduced to reduce NO_x_ emissions of diesel engine (Beale et al., 2015).

## Future Directions

Although mechanical processing gives rise to relatively fast and inexpensive results, the small size of tobacco seeds leads to relatively high requirements for pressing equipment and pretreatment technology, which can subsequently raise production costs. The advantages of conventional extraction with solvents are low cost, simple equipment, no need for filtration, and high efficiency, but the disadvantages are environmental pollution and the application of high temperatures (Castro and Priego-Capote, 2010). The application of the supercritical fluid extraction process is based on the balance between economic benefits and environmental benefits. Recently, new extraction processes using renewable natural products or nonhazardous solvents have been applied to extractions of grape seed oil and sunflower oil (Sineiro et al., 1998; Passos et al., 2009). Enzyme extraction is an enzyme-assisted aqueous extraction that operates under moderate conditions and provides a high oil yield. In the future, processes for the extraction of tobacco seed oil should combine two or more extraction techniques into a complete process or modify available techniques to enhance oil yields. Moreover, more economical and environmentally friendly extraction processes should be utilized.

The ideal biodiesel transesterification catalyst should have the characteristics of high catalytic capacity, stability, minimal lixiviation, reusability, low cost, recyclability, etc. These are crucial for the selection of appropriate catalysts and reaction conditions in biodiesel production from tobacco seed oil. Heterogeneous catalysts are easy to separate from the reaction mixture, regenerate and reuse. Therefore, heterogeneous catalysts will likely replace traditional homogeneous catalysts for transesterification of tobacco seed oil in the near future. Compared with other transesterification methods, the enzymatic method has the following advantages: the catalyst (i.e., enzyme) is environmentally friendly; the operating conditions are moderate; the quality of the by-product glycerin is high; side effects such as saponification are few; the purity of the final product is high, and the enzyme method allows a relatively high FFA content and proceeds with one step. Hence, the enzymatic production of biodiesel is another important direction for the development of tobacco seed oil use. Although the catalysts for these two methods are relatively slow and expensive, novel heterogeneous and enzymatic catalysis may overcome these drawbacks.

Biotechnology, such as overexpression, silencing, knockout, and editing of genes associated with seed development, plays vital roles in the accumulation and production of tobacco seed oil in the future. Using the mining of genes and proteins involved in tobacco seed oil biosynthesis, the content of tobacco seed oil will be increased through metabolic pathways. Reorganization of acyl flux and coexpression of genes involved in lipid metabolism and TAG production accumulated more than 15% oil (dry weight) in tobacco leaves (Vanhercke et al., 2014). Tobacco seed oil contents differ among different tobacco varieties, so the yield of biodiesel from tobacco seeds can be increased by cultivating high-oil varieties. Additionally, grafting may also be an important way to increase biodiesel yields from tobacco seeds during planting. Strikingly, national policies and government support will also affect the development and research of tobacco seed oil and leaves for the production of biodiesel.

Recently, many countries have implemented higher standards and requirements for biodiesel fuel characteristics and pollution emissions. Further research should focus on modifications of TSOBD properties to meet diesel standards and assess the efficiency of TSOBD in an experimental engine setup, either alone or blended with diesel. The evaluation of biodiesel should involve all of the parameters specified in standards and provide for a good balance between engine performance and NO_*x*_ emissions by optimizing engine operating parameters or designing new diesel engines.

## Conclusions

Tobacco seed is identified as a promising feedstock for sustainable biodiesel production due to its suitable physicochemical composition, higher oil yields, and lower competition with food crops. However, securing sustainable and sufficient amounts of tobacco seed oil for large scale biodiesel production is not achieved yet. The tobacco seed for sustainable biodiesel production is significantly affected by various factors, which could be considered as varieties, cultivation, extraction, transesterification, application etc. Thus, future studies should focus on the higher-oil varieties with biotechnology, high-effective cultivation technique, economical and environmentally friendly extraction methods, novel heterogeneous or enzymatic catalyzed transesterifications and modifications of TSOBD physicochemical properties.
